# Computational Neuroscience Ontology: a new tool to provide semantic meaning to your models

**DOI:** 10.1186/1471-2202-13-S1-P149

**Published:** 2012-07-16

**Authors:** Yann Le Franc, Andrew P Davison, Padraig Gleeson, Fahim T Imam, Birgit Kriener, Stephen D Larson, Subhasis Ray, Lars Schwabe, Sean Hill, Erik De Schutter

**Affiliations:** 1 Dept. of Biomedical Sciences, University of Antwerp, Wilrijk, Belgium; 2 UNIC, Centre National de la Recherche Scientifique, Gif sur Yvette, France; 3 Dept. of Neuroscience, Physiology and Pharmacology, University College London, UK; 4 Neuroscience Information Framework, University of California, San Diego, USA; 5 Dept. of Mathematical Sciences and Technology. Norwegian University of Life Sciences, Ås, Norway; 6 National Centre for Biological Sciences, Bangalore, India; 7 Dept. of Computer Science and Electrical Engineering, University of Rostock, Rostock, Germany; 8 International Neuroinformatics Coordinating Facility, Stockholm, Sweden; 9 Computational Neuroscience Unit, Okinawa Institute of Science and Technology, Onna, Japan

## 

The diversity of modeling approaches in computational neuroscience makes model sharing, retrieval, reuse and reproducibility difficult and even sometimes impossible. To address this problem, standardized languages have been developed by and for the community, such as NeuroML[1], PyNN [2] and NineML (http://software.incf.org/software/nineml). Although these languages enable software interoperability and therefore model reuse and reproducibility, they lack semantic information that would facilitate efficient model sharing and retrieval.

In the context of the INCF Multi-Scale Modeling (MSM) program, we have developed an ontology to annotate spiking network models described with NineML and other structured model description languages. Ontologies are formal models of knowledge in a particular domain and composed of classes that represent concepts defining the field as well as the logical relations that link these concepts together [3]. These classes and relations have unique identifiers and definitions that allow unambiguous annotation of digital resources such as web pages or model source code. Implemented in a machine-readable format, these knowledge models can be used to design more efficient and intuitive information retrieval systems for experts in the field.

We are proposing the first version of the Computational Neuroscience Ontology or CNO. This ontology is composed of 207 classes representing general concepts related to computational neuroscience organized in a hierarchy of concepts. CNO is currently available on Bioportal (http://bioportal.bioontology.org/ontologies/3003).

The design of CNO follows some of the recommendations of the Open Biological and Biomedical Ontologies (OBO) community and is compatible with the ontologies developed and maintained within the Neuroscience Information Framework (NIF, [4]http://www.neuinfo.org). Integration with this large federation of neuroscience ontologies has two main advantages: (1) it allows the linking of models with biological information, creating a bridge between computational and experimental knowledge bases; (2) as ontology development is an iterative process that relies on inputs from the community, NIF has developed NeuroLex (http://neurolex.org), an effective collaborative platform, available for community inputs on the content in CNO.

With the further development of CNO based on inputs from the community, we hope CNO will provide a useful framework to federate digital resources in the field of computational neuroscience.
